# Use and Perceptions of Teletherapy Among Physiotherapists in Germany: Descriptive Cross-Sectional Online Survey

**DOI:** 10.2196/93733

**Published:** 2026-09-21

**Authors:** Tobias Michels, Christoff Zalpour, Bernhard Elsner

**Affiliations:** 1 Faculty of Economics and Social Sciences, Hochschule Osnabrück, Albrechtstraße 30, Osnabrück, Lower Saxony, 49076, Germany, 49 0541 969-7241; 2 Institute for Health Sciences, University of Lübeck, Lübeck, Schleswig-Holstein, Germany; 3 Fraunhofer Research Institution for Individualized Medical Technology and Engineering IMTE, Lübeck, Schleswig-Holstein, Germany

**Keywords:** physiotherapy telehealth, digital health implementation, barriers and facilitators, cross-sectional survey, health services research

## Abstract

**Background:**

Teletherapy is gaining increasing importance in physiotherapeutic care but has so far been only marginally established in Germany. The current state of use, attitudes, and perceived barriers among physiotherapists remains insufficiently described.

**Objective:**

This online survey aimed to assess perceived barriers, enabling factors, and competence-related needs for the implementation and use of physiotherapeutic teletherapy among participating physiotherapists in Germany.

**Methods:**

A descriptive cross-sectional online survey was conducted among practicing physiotherapists in Germany using the LimeSurvey platform between March 7 and May 21, 2025. Participants were recruited through an open convenience sampling strategy via physiotherapy-related social media channels, university alumni mailing lists, the mailing list of the German Association for Musculoskeletal Therapy, and an article on physio.de; all individuals with access to the survey link were able to participate. The questionnaire collected demographic and professional data as well as information on current and intended use of teletherapy, perceived barriers and benefits, training needs, and self-assessed competence to deliver teletherapy. Data were analyzed descriptively.

**Results:**

A total of 761 individuals started the survey, of whom 458 completed the questionnaire in full and were included in the analysis. In this sample, 30 participants reported prior experience with teletherapy. Most participants considered teletherapy meaningful for future physiotherapy care, but only a minority felt sufficiently informed about its use. Frequently reported barriers included integration into daily practice routines, insufficient information on legal and organizational frameworks, and insufficient digital infrastructure within practices. Participants most frequently identified further training, exchange and support from colleagues, and information from professional associations and health insurance providers as potential facilitators. Exploratory descriptive stratified analyses suggested that participants with prior teletherapy experience more often felt sufficiently informed and competent and reported selected implementation barriers less frequently than participants without prior experience.

**Conclusions:**

In this sample, physiotherapeutic teletherapy was rarely used but was widely perceived as meaningful for future care. Participants reported structural, organizational, informational, and competence-related barriers that may limit routine implementation. These findings suggest that clearer guidance, targeted training, and implementation support from professional associations, health insurance providers, and educational institutions may help physiotherapists integrate teletherapy into practice where clinically appropriate.

## Introduction

The increasing demands within Western health care systems, driven by demographic change, rising multimorbidity, and a shortage of skilled professionals [1,2], make it necessary to establish more efficient models of care within physiotherapy, particularly in Germany. Physiotherapeutic teletherapy offers one such opportunity but remains insufficiently explored within the German health care system. Physiotherapeutic teletherapy usually refers to remote physiotherapy services delivered without physical copresence, particularly video-based or otherwise digitally mediated therapeutic encounters. Telerehabilitation usually refers to a broader rehabilitation-oriented concept, whereas digital health is commonly used as an umbrella term for wider digital technologies and services in health care. Existing evidence suggests that the implementation of video-based telehealth in allied health professions is influenced by a broad range of barriers at technological, organizational, professional, interpersonal, patient-related, and regulatory levels. A scoping review by Rettinger and Kuhn [3] identified technology issues, practice issues, patient issues, environmental issues, attributions, interpersonal issues, policies and regulations, and administration issues as central barrier categories among allied health professionals and nurses. Frequently reported barriers included the lack of hands-on experience, unreliable network connections, limited access to technology, reduced quality of visual observation and instruction, lack of technology skills, and impaired client-practitioner interaction and communication [3]. Evidence specific to physiotherapy points to similar challenges. In a mixed methods study of German physiotherapists, Stahn et al [4] found that telerehabilitation use peaked during the COVID-19 lockdowns but declined afterward, suggesting that pandemic-related adoption did not necessarily translate into stable routine use. The most prominent barrier reported in that study was the lack of physical examination, while qualitative findings additionally highlighted concerns regarding technical infrastructure, legal ambiguity, professional skepticism, and the need for structured implementation processes [4]. At the same time, facilitators included the promotion of patient self-management, broadening therapy options, appropriate technical infrastructure, structured implementation, and patient involvement in decision-making [4]. Existing studies on teletherapy in German physiotherapy are limited and often context-specific, for example, focusing on the COVID-19 pandemic [5] or geriatric patients [6]. A recent survey of 300 physiotherapy practices in Germany reported that only 1% currently provide teletherapy services, indicating that teletherapy use among physiotherapists in Germany remains highly limited [7]. At the same time, current political developments indicate a national effort to strengthen digitalization in German health care. Legislative initiatives such as the Act to Accelerate the Digitalisation of the Healthcare System [8] and the Act to Improve the Use of Health Data [9], governmental subsidies for teletherapy-related hardware and software [10], the planned connection of physiotherapy practices to the telematics infrastructure [11], and the nationwide rollout of the electronic patient record [12] highlight the need for physiotherapy to engage with digitalization. However, the extent to which physiotherapists perceive teletherapy as feasible, useful, and implementable in routine care remains insufficiently described. This online survey provides an initial descriptive assessment among participating physiotherapists in Germany, aiming to gather information on perceived barriers, enabling factors, and competencies required for the implementation and use of physiotherapeutic teletherapy.

## Methods

### Study Design

This online survey was conducted between March 7, 2025, and May 21, 2025. The target population comprised all physiotherapists practicing in Germany who had completed professional training. The total number of physiotherapists in Germany is estimated to be approximately 200,000 [13]. The estimated size of the target population was based on publicly available professional statistics from Physio Deutschland; no individual registry of practicing physiotherapists was used for recruitment. Based on this population size and assuming a 95% CI and a margin of error of ±5%, the required sample size was calculated to be approximately 384. As no reliable prior estimate was available for the expected proportion of physiotherapists reporting key survey outcomes, such as perceived barriers, facilitators, or previous teletherapy experience, a proportion parameter of *P*=.5 was used. This value represents the most conservative assumption for a proportion estimate because it maximizes variance and therefore yields the largest required sample size. The questionnaire was administered using the LimeSurvey platform and conducted as an open survey. The survey was accessible through a standard web browser and was mobile-friendly, allowing completion on desktop and mobile devices. Cookies were used to prevent or reduce multiple participation. To ensure transparent and comprehensive reporting of the online survey, the CHERRIES (Checklist for Reporting Results of Internet E-Surveys) was applied [14]. The reporting addressed CHERRIES items including survey design, ethics approval and informed consent, data protection, recruitment and sampling, survey administration, prevention of multiple entries, completeness checks, and analysis of complete questionnaires. Deviations from CHERRIES recommendations mainly related to the open convenience sampling strategy because the survey link was distributed through open professional and academic channels; the number of individuals who received or viewed the invitation could not be determined, and no conventional response rate could be calculated. The completed CHERRIES checklist is provided as Checklist 1. The study was preregistered in the Center for Open Science database.

### Recruitment and Sampling

Participants were recruited using an open convenience sampling strategy. Individuals were not identified or contacted through a predefined sampling frame; instead, the survey link was disseminated through physiotherapy-related professional and academic channels. Recruitment took place via the social media channels of the German Society for Physiotherapy Science (DGPTW), alumni mailing lists of the physiotherapy degree programs at Osnabrück University of Applied Sciences and Bremen University of Applied Sciences, the email distribution list of the German Association for Musculoskeletal Therapy (DVMT), and an article published on physio.de. The survey was accessible to all individuals who had access to the survey link. Because recruitment was conducted through open dissemination channels, the number of individuals who received or viewed the invitation could not be determined, and no conventional response rate could be calculated.

### Questionnaire

Although instruments related to teletherapy and telehealth have been developed, they did not adequately match the aim and target population of this study. The Teletherapy Intervention Scale was developed to assess therapeutic alliance and presence in telepsychotherapy and therefore does not address the specific organizational, regulatory, technical, and competence-related conditions of physiotherapeutic teletherapy [15]. Similarly, the Telehealth Competency Questionnaire-Provider assesses general provider competencies in telehealth, but is not specific to physiotherapy, the German health care context, or the implementation barriers and facilitators relevant to physiotherapy practices [16]. Therefore, a study-specific questionnaire was developed to assess current and intended use, perceived barriers and benefits, training needs, and self-assessed competence among physiotherapists in Germany. It addressed the following domains:

1. Previous experiences with teletherapy
2. Challenges and barriers
3. Facilitators and enabling factors
4. Competencies required for teletherapy use
5. Demographic characteristics

The questionnaire consisted of 23 items and took approximately 10 minutes to complete. It was administered in German. No translation or back-translation procedures were required for data collection because the target population consisted of physiotherapists practicing in Germany. The complete questionnaire is provided as Multimedia Appendix 1. The survey focused on professional, organizational, and implementation-related factors relevant to physiotherapeutic teletherapy. To keep the questionnaire brief and reduce the collection of personal data, sex was not included as a demographic item. We acknowledge that sex may be relevant for describing the sample and for examining potential equity- and diversity-related differences in perceptions, barriers, and implementation needs. The initial item pool was developed by the authors based on the study objectives, the domains identified as relevant for teletherapy implementation, existing literature on telehealth and telerehabilitation barriers and facilitators, and the German health care context. Items were designed to capture previous teletherapy experience, perceived barriers and concerns, facilitators and support needs, self-assessed competence, and demographic and professional characteristics. A pretest was carried out with 5 physiotherapists who had expertise in digital health care and questionnaire development. The pretest was used to assess comprehensibility, relevance, feasibility, wording, response options, and estimated completion time. Based on the pretest feedback, minor revisions were made to improve wording, clarify item instructions, and refine response options. No formal psychometric validation or reliability testing was conducted. Most questions were multiple-choice items, and all thematic sections included optional open-text fields allowing participants to provide individualized responses when predefined options were insufficient. Category D, “Competencies required for physiotherapeutic teletherapy,” was assessed using an open-ended response format. These qualitative data are not reported in this paper and will be analyzed and published separately.

### Data Analysis

Data were analyzed using IBM SPSS Statistics (version 28.0.1.0). Only fully completed questionnaires were included in the analysis. Incomplete questionnaires were excluded because the study aimed to provide a consistent descriptive overview across all survey domains, and the main analyses required complete information across the questionnaire sections. Alternative approaches, such as item-level analyses with varying denominators or imputation of missing responses, were not used because they would have produced inconsistent denominators across survey domains and would not have addressed the systematic absence of later questionnaire sections among participants who discontinued the survey before completion. No data imputation was performed. To assess potential attrition, available individual-level partial responses from incomplete questionnaires were reviewed descriptively. This included survey progress information and available responses to items that had been reached before dropout. Because demographic characteristics were collected at the end of the questionnaire, they were largely unavailable for incomplete questionnaires. Therefore, available partial responses from noncompleters were used only for a descriptive attrition assessment and were not included in the main analysis. All datasets were checked for inconsistencies prior to analysis. As the study aimed to provide an exploratory and descriptive overview of teletherapy use and perceptions among participating physiotherapists, no inferential statistical analyses were conducted for the primary analyses. For categorical variables, absolute and relative frequencies (n, %) were calculated. For multiple-choice questions that included an “other” response option, free-text entries were reviewed descriptively and counted as additional response frequencies where applicable. Exploratory descriptive post hoc stratified analyses were conducted for selected implementation-relevant outcomes. Outcomes included perceived meaningfulness of teletherapy for future health care delivery, feeling sufficiently informed, self-assessed competence to offer teletherapy, selected key barriers, and selected key facilitators. Results were stratified by reported teletherapy experience or use, self-rated technical proficiency, and professional experience. For the stratified analyses, teletherapy experience was defined as any self-reported previous or current use of physiotherapeutic teletherapy. No minimum threshold for frequency, duration, number of patients, or regularity of use was applied. Therefore, participants who reported any prior teletherapy experience were grouped as having teletherapy experience, whereas participants without any reported previous or current use were grouped as having no teletherapy experience. Professional experience was dichotomized into 10 years or less and more than 10 years. These analyses were descriptive only, and no inferential statistical testing was performed.

### Ethical Considerations

The study protocol was reviewed and approved by the Ethics Committee of Osnabrück University of Applied Sciences prior to data collection (HSOS2024/1/15). All participants received information about the aim of the study, data protection, data storage, and data processing before starting the survey. Participation was voluntary, and informed consent was obtained electronically before participants proceeded to the questionnaire. The survey did not collect directly identifying personal information, and data were analyzed anonymously. Data were processed in accordance with the General Data Protection Regulation (GDPR) and stored on a password-protected institutional laptop. No incentives or participant compensation were offered.

## Results

### Sociodemographic and Professional Characteristics

**Figure 1.**
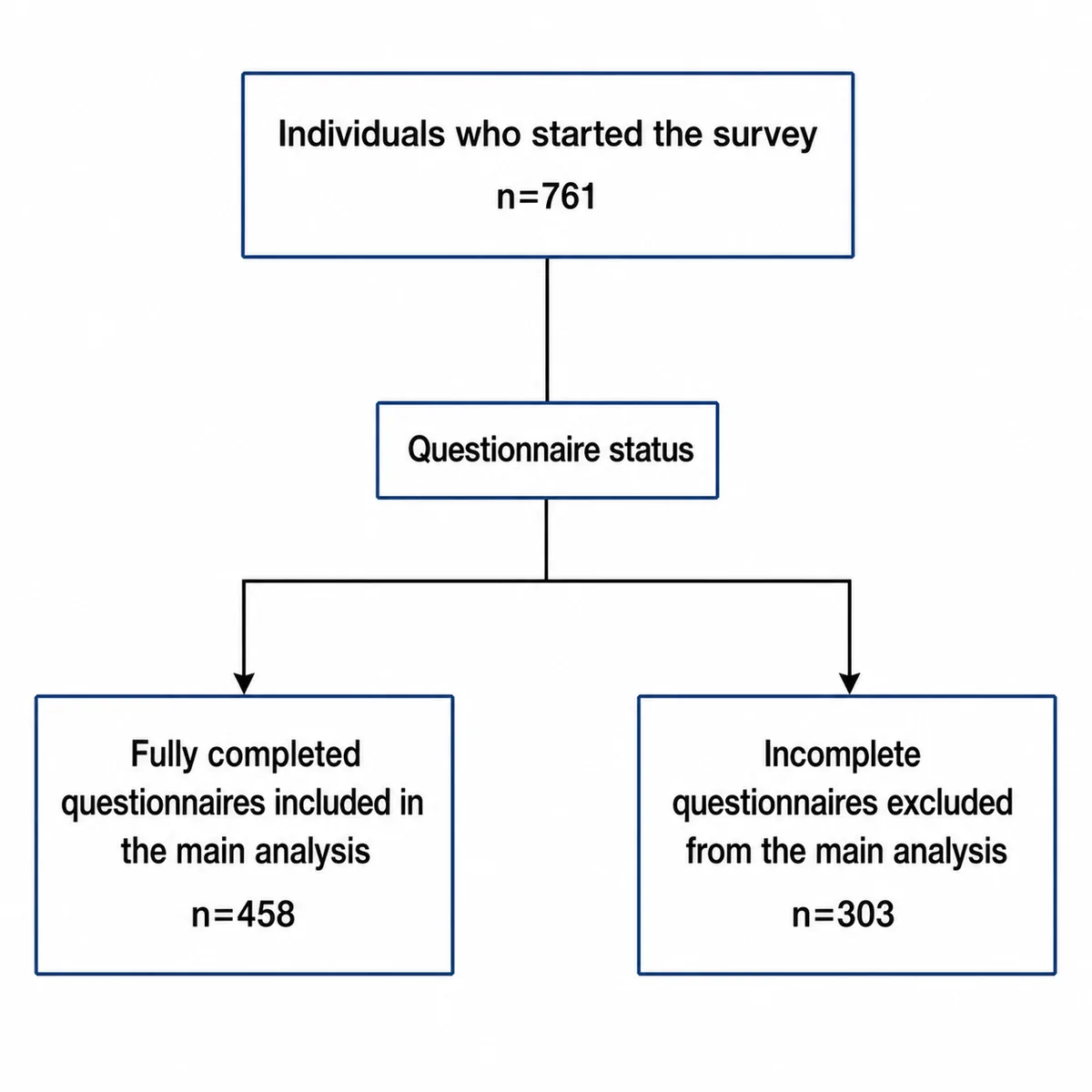
Participant flow through the online survey.

A total of 761 participants started the survey, of whom 458 (60.18%) completed the questionnaire in full and were included in the analysis (Figure 1).

Among the 303 incomplete questionnaires, 112 participants did not progress beyond the initial survey page, 125 reached page 1, 58 reached page 2, and 8 reached page 3. Among noncompleters who answered the initial item on previous teletherapy use, 4 of 192 (2.1%) reported previous teletherapy use, compared with 30 of 458 (6.6%) completers. Among the 71 noncompleters who reached the main barriers section, the most frequently selected barriers were integration into daily practice routines (38/71, 53.5%), insufficient information on legal and organizational frameworks (32/71, 45.1%), and insufficient digital infrastructure within practices (29/71, 40.8%). This pattern was broadly similar to the corresponding findings among completers. Because demographic characteristics were collected at the end of the questionnaire and were almost entirely unavailable for noncompleters, no meaningful demographic comparison between completers and noncompleters could be conducted. Participants’ ages ranged from 20 to 74 years, with a mean age of 34 (SD 11.20) years. The average professional experience was 12 (range 1‐50) years. Sociodemographic and professional characteristics of the sample are summarized in Table 1. Sex was not assessed in the questionnaire. The distribution of educational qualifications, practice location by city size, selected therapeutic specialization, technical proficiency, and prior experience with teletherapy is presented in Table 1. Approximately one-third of the participants practiced in a city with 100,000 to 500,000 inhabitants, and nearly one-fifth worked in a large metropolitan area with more than 500,000 inhabitants. The most frequently reported therapeutic specialization was treatment of a mixed clientele (195/458, 42.58%), followed by orthopedic patients (159/458, 34.72%). A total of 72.05% (330/458) of participants rated themselves as either very technically proficient or somewhat technically proficient.

**Table 1.** Sociodemographic and professional characteristics of participants^a^.

Characteristic	Value
Survey participation, n (%)	
Individuals who started the survey	761 (100)
Fully completed questionnaires included in analysis	458 (60.18)
Incomplete questionnaires excluded	303 (39.82)
Age (years)	
Mean (SD)	34 (11.2)
Range	20‐74
Sex	Not assessed
Professional experience (years)	
Mean	12
Range	1‐50
Educational qualification, n (%)	
State examination	271 (59.17)
Bachelor’s degree	125 (27.29)
Master’s degree	56 (12.23)
Doctorate	6 (1.31)
Practice location by city size, n (%)	
<5000 inhabitants	33 (7.21)
5000‐10,000 inhabitants	31 (6.77)
>10,000‐20,000 inhabitants	59 (12.88)
>20,000‐50,000 inhabitants	51 (11.14)
>50,000‐100,000 inhabitants	46 (10.04)
>100,000‐500,000 inhabitants	155 (33.84)
>500,000 inhabitants	83 (18.12)
Selected therapeutic specialization, n (%)	
Mixed clientele	195 (42.58)
Orthopedic patients	159 (34.72)
Self-rated technical proficiency, n (%)	
Very or somewhat technically proficient	330 (72.05)
Previous experience with teletherapy, n (%)	
Any previous teletherapy experience	30 (6.55)
Teletherapy use on more than 5 d/mo	6 (1.31)

a Unless otherwise stated, percentages refer to the final analysis sample (N=458). Percentages for survey participation refer to all individuals who started the survey (n=761). Sex was not assessed. Selected therapeutic specialization is reported for the most frequently selected categories.

### Previous Experience With Physiotherapeutic Teletherapy

A total of 30 (6.55%) participants reported having prior experience with teletherapy. Among these, 6 (1.32%) individuals stated that they used or had used teletherapy on more than 5 days per month. The most frequently reported number of patients receiving teletherapy per month was 1‐5 patients (n=18). Zoom (Zoom Communications, Inc) was identified as the most commonly used platform for conducting teletherapy sessions (n=12), while all other software solutions were mentioned only sporadically. Regarding the reasons for prior teletherapy use among participants with previous experience, the most frequently selected responses were service for patients (n=22), personal motivation (n=14), and preparation for the future (n=13). Multiple responses were possible. Participants without previous teletherapy experience were asked what reasons would motivate them to use teletherapy in the future. The most frequently selected responses were service for patients (285/458, 62.23%), preparation for the future (223/458, 48.69%), and personal motivation (212/458, 46.29%). Detailed results on motivations for future use are summarized in Table 2.

**Table 2.** Motivations, facilitators, support needs, and perceived advantages of physiotherapeutic teletherapy^a^.

Domain or item	Value, n (%)
Reasons that would motivate future teletherapy use among participants without previous teletherapy experience	
Service for patients	285 (62.23)
Preparation for the future	223 (48.69)
Personal motivation	212 (46.29)
Financial incentive	108 (23.58)
No reason	57 (12.45)
Other reasons	35 (7.64)
Factors that would support future teletherapy provision	
Further training	272 (59.39)
Exchange and support from colleagues	248 (54.15)
Information from professional associations	215 (46.94)
Information from health insurance providers	190 (41.48)
Social media	136 (29.69)
Other support factors	48 (10.48)
Stakeholders expected to provide relevant information	
Professional associations	361 (78.82)
Health insurance providers	281 (61.35)
Software providers	234 (51.09)
Federal and state governments	156 (34.06)
Other stakeholders	25 (5.46)
Perceived advantages of teletherapy	
Expanded service for patients, including positive marketing	324 (70.74)
Reduced infection risk for patients and therapists	261 (56.99)
Reduced need for home visits	180 (39.30)
Reduction of ecological footprint	179 (39.08)
Possibility of treating more patients in the same time frame	103 (22.49)
Other advantages	69 (15.07)

a Percentages refer to the final analysis sample (N=458). Multiple responses were possible. The domain “Reasons that would motivate future teletherapy use” refers to hypothetical future motivations among participants without previous teletherapy experience.

### Barriers and Concerns Related to Physiotherapeutic Teletherapy

The most frequently reported barrier to the use or planned use of physiotherapeutic teletherapy was integration into daily practice routines (274/458, 59.83%). This was followed by insufficient information on legal and organizational frameworks (220/458, 48.03%), insufficient digital infrastructure within practices (191/458, 41.70%), and a lack of professional knowledge regarding the implementation of teletherapy (170/458, 37.12%). Less frequently reported barriers included a lack of technical knowledge (106/458, 23.14%), insufficient internet connectivity (50/458, 10.92%), and no perceived added value (63/458, 13.76%). All barriers and concerns are summarized in Table 3. Participants were also asked what generally speaks against the use of physiotherapeutic teletherapy. The most frequently selected concerns were lower treatment quality compared with in-person care (228/458, 49.78%) and the assumption that patients do not want digital treatments (222/458, 48.47%). Other concerns included impractical health insurance regulations (147/458, 32.10%), a perceived high risk of inadequate care (141/458, 30.79%), the need for direct physical contact between patients and therapists (106/458, 23.14%), and insufficient available software solutions (86/458, 18.78%). Multiple responses were possible.

**Table 3.** Barriers and concerns related to physiotherapeutic teletherapy^a^.

Domain or item	Value, n (%)
Barriers and challenges to implementation	
Integration into daily practice routines	274 (59.83)
Insufficient information on legal and organizational frameworks	220 (48.03)
Insufficient digital infrastructure within practices	191 (41.70)
Lack of professional knowledge regarding implementation	170 (37.12)
Lack of patient interest	138 (30.13)
Lack of technical knowledge	106 (23.14)
No perceived added value	63 (13.76)
Insufficient internet connectivity	50 (10.92)
Other barriers	38 (8.30)
Concerns about teletherapy use	
Lower treatment quality compared with in-person care	228 (49.78)
Assumption that patients do not want digital treatments	222 (48.47)
Impractical health insurance regulations	147 (32.10)
Perceived high risk of inadequate care	141 (30.79)
Patients require direct physical contact with therapists	106 (23.14)
Insufficient available software solutions	86 (18.78)
Other concerns	67 (14.63)

a Percentages refer to the final analysis sample (N=458). Multiple responses were possible.

### Facilitators, Support Needs, and Perceived Advantages

Participants were asked whether they considered physiotherapeutic teletherapy to be meaningful for future health care delivery. The majority of respondents agreed that teletherapy represents a useful approach for future physiotherapy services (362/458, 79.04%), whereas 20.96% (96/458) did not. However, only a minority of participants reported feeling sufficiently informed about the possibility of using physiotherapeutic teletherapy (83/458, 18.12%), whereas the majority did not feel sufficiently informed (375/458, 81.88%). In addition, 38.43% (176/458) of participants stated that they felt competent enough to work with teletherapy. The most frequently reported factors that would support participants in offering physiotherapeutic teletherapy in the future were further training (272/458, 59.39%), exchange and support from colleagues (248/458, 54.15%), information from professional associations (215/458, 46.94%), and information from health insurance providers (190/458, 41.48%). Participants most frequently expected professional associations (361/458, 78.82%) and health insurance providers (281/458, 61.35%) to be responsible for preparing and providing relevant information, followed by software providers (234/458, 51.09%) and federal and state governments (156/458, 34.06%). Participants were also asked about the general advantages of physiotherapeutic teletherapy. The most frequently reported benefit was an expanded service for patients, including positive marketing (324/458, 70.74%). More than half of the respondents highlighted a reduced risk of infection for both patients and therapists (261/458, 56.99%). Further perceived advantages included reduced need for home visits (180/458, 39.30%), reduction of the ecological footprint (179/458, 39.08%), and the possibility of treating a higher number of patients within the same time frame (103/458, 22.49%). Motivations, facilitators, expected sources of support, and perceived advantages are summarized in Table 2.

### Exploratory Descriptive Stratified Analyses

The following subgroup findings are exploratory, descriptive, and hypothesis-generating only. No inferential statistical testing was performed. Participants with reported teletherapy experience or use (n=30) more frequently considered teletherapy meaningful for future health care delivery than those without such experience (29/30, 96.7% vs 333/428, 77.8%). They also more often reported feeling sufficiently informed (16/30, 53.3% vs 67/428, 15.7%) and competent enough to offer teletherapy (22/30, 73.3% vs 154/428, 36%). In contrast, barriers such as integration into daily practice routines, insufficient legal or organizational information, and insufficient digital infrastructure were reported less frequently by participants with teletherapy experience or use. Participants who rated themselves as very or somewhat technically proficient (n=330) more often considered teletherapy meaningful for future care than participants with neutral or lower technical proficiency (270/330, 81.8% vs 92/128, 71.9%). They also more frequently reported feeling competent enough to offer teletherapy (138/330, 41.8% vs 38/128, 29.7%). Differences between these groups were smaller for feeling sufficiently informed and for the most frequently reported barriers. Professional experience showed a different pattern. Participants with 10 years or less of professional experience (n=262) more often considered teletherapy meaningful for future care (224/262, 85.5% vs 138/196, 70.4%), but less often reported feeling sufficiently informed (25/262, 9.5% vs 58/196, 29.6%) or competent enough to offer teletherapy (83/262, 31.7% vs 93/196, 47.4%) compared with participants with more than 10 years of experience (n=196). Less experienced participants also reported barriers such as integration into daily practice routines, insufficient legal or organizational information, and insufficient digital infrastructure more frequently. The full descriptive stratified results are presented in Table 4.

**Table 4.** Exploratory descriptive stratified analyses of selected implementation-relevant outcomes^a^.

Outcome	TT exp^b^ (n=30)	No TT exp (n=428)	High tech proficiency^c^ (n=330)	Lower tech proficiency^d^ (n=128)	≤10 yrs exp^e^ (n=262)	>10 yrs exp (n=196)
Meaningfulness and competence, n (%)						
Teletherapy meaningful for future care	29 (96.67)	333 (77.80)	270 (81.82)	92 (71.88)	224 (85.50)	138 (70.41)
Felt sufficiently informed	16 (53.33)	67 (15.65)	63 (19.09)	20 (15.63)	25 (9.54)	58 (29.59)
Felt competent to offer teletherapy	22 (73.33)	154 (35.98)	138 (41.82)	38 (29.69)	83 (31.68)	93 (47.45)
Selected barriers, n (%)						
Integration into daily routines	7 (23.33)	267 (62.38)	205 (62.12)	69 (53.91)	183 (69.85)	91 (46.43)
Insufficient legal or organizational information	9 (30.00)	211 (49.30)	162 (49.09)	58 (45.31)	146 (55.73)	74 (37.76)
Insufficient digital infrastructure	4 (13.33)	187 (43.69)	135 (40.91)	56 (43.75)	136 (51.91)	55 (28.06)
Selected facilitators, n (%)						
Further training	7 (23.33)	265 (61.92)	193 (58.48)	79 (61.72)	185 (70.61)	87 (44.39)
Peer exchange and support	13 (43.33)	235 (54.91)	184 (55.76)	64 (50.00)	166 (63.36)	82 (41.84)

a Values are n (%). Percentages refer to the respective subgroup shown in the column heading. Analyses were exploratory, descriptive post hoc analyses; no inferential statistical testing was performed. For barriers and facilitators, multiple responses were possible.

b TT exp: teletherapy experience or use.

c High tech proficiency: very or somewhat technically proficient.

d Lower tech proficiency: neutral or lower technical proficiency.

e yrs exp: years of professional experience.

## Discussion

### Principal Findings

This cross-sectional online survey provides a current descriptive assessment of participating physiotherapists’ perceptions of teletherapy in Germany, focusing on previous use, perceived barriers and benefits, support needs, and self-assessed competence. The findings show that previous experience with physiotherapeutic teletherapy was low in this sample, with only 6.55% (30/458) of participants reporting prior use. At the same time, most respondents considered teletherapy meaningful for future physiotherapy care. This indicates a gap between perceived relevance and reported routine implementation among participating physiotherapists. Reported barriers and facilitators clustered around practical implementation rather than conceptual rejection of teletherapy. Participants described uncertainty about how teletherapy can be integrated into daily routines, legally and organizationally framed, technically supported, and delivered with sufficient competence. This pattern suggests that implementation support may need to focus less on general awareness of teletherapy and more on practical guidance, training, workflow integration, and clarification of regulatory and organizational conditions. Taken together, these findings suggest that limited implementation in this sample may not be primarily explained by rejection of teletherapy as a concept, but by uncertainty about how teletherapy can be safely, legally, technically, and practically integrated into everyday physiotherapy care.

### Comparison With Previous Research

The low level of previous teletherapy experience in the present sample is consistent with existing evidence indicating that teletherapy use in German physiotherapy remains limited. Hambloch et al [7] reported that only 1% of 300 surveyed physiotherapy practices in Germany currently provided teletherapy services. Although the proportion of participants with prior teletherapy experience was higher in this study, the overall pattern remains similar: teletherapy appears to be recognized as potentially relevant by many respondents but has not yet become a routine component of physiotherapy practice in Germany. The findings also align with the broader implementation literature on video-based telehealth in allied health professions. Rettinger and Kuhn [3] identified technology issues, practice issues, patient issues, environmental issues, attributions, interpersonal issues, policy and regulation issues, and administration issues as major categories of barriers among allied health professionals and nurses. Several of these categories were reflected in the present study. For example, insufficient digital infrastructure and internet connectivity correspond to technology-related barriers, while difficulty integrating teletherapy into daily practice routines reflects practice and organizational barriers. Concerns about treatment quality, patient acceptance, and the lack of direct physical contact also correspond to barriers related to clinical practice, patient issues, and professional perceptions. Evidence from musculoskeletal and primary health care contexts points to similar implementation challenges. In a systematic review on digital health services for people with musculoskeletal conditions in primary health care, van Tilburg et al [17] identified barriers and facilitators related to workflow integration, technology and infrastructure, organizational and regulatory conditions, training, and clear implementation procedures. This supports the interpretation that several barriers reported in the present sample, particularly integration into daily practice routines, insufficient digital infrastructure, insufficient legal or organizational information, and the need for further training, are consistent with broader implementation challenges in digital health services for musculoskeletal care. Evidence specific to German physiotherapy further supports these findings. Stahn et al [4] reported that telerehabilitation use among German physiotherapists increased during COVID-19 lockdowns but declined afterward, suggesting that pandemic-related adoption did not necessarily translate into sustained routine implementation. Their study identified lack of physical examination as a major barrier and highlighted concerns about technical infrastructure, legal ambiguity, professional skepticism, and the need for structured implementation processes [4]. The present findings extend this evidence by showing similar perceived barriers in a larger national sample and by quantifying the extent to which participating physiotherapists reported insufficient information, limited self-assessed competence, and a need for training and professional support. International clinician perspectives from the COVID-19 era point in a similar direction. In a survey among allied health clinicians treating musculoskeletal conditions, Malliaras et al [18] reported increased telehealth use during the pandemic but also persistent concerns about the limited hands-on nature of telehealth, clinical suitability, patient resources, and provider capability or training. These findings align with the present results, in which participants reported concerns about treatment quality, the need for direct physical contact, patient willingness, and further training needs. International survey evidence from physiotherapy supports the interpretation that several barriers identified in this study are part of broader implementation challenges rather than being specific to Germany alone. In a web-based survey among 338 primary care physiotherapists treating patients with musculoskeletal disorders in Norway, Martinsen et al [19] found that almost half of respondents had adopted digital health technologies in clinical practice and that physiotherapists generally reported positive attitudes toward their use. However, concerns were also reported regarding the lack of physical examination, technical aspects, and the need for training in the use of digital health technologies [19]. These findings correspond to the present results, in which participants reported concerns about reduced treatment quality, the need for direct physical contact, insufficient digital infrastructure, and further training as an important facilitator. At the same time, the higher reported adoption in the Norwegian survey may indicate that implementation is influenced by health care system context, digital maturity, and the broader definition of digital health technologies used in that study, which included more than video-based teletherapy alone [19]. Overall, the comparison with previous research suggests that some barriers reported in this study reflect broader international implementation challenges in telehealth and digital health, including workflow integration, technical infrastructure, clinical suitability, patient acceptance, and training needs. In contrast, uncertainty regarding reimbursement, legal and organizational frameworks, connection to the telematics infrastructure, and the role of German health insurance providers reflects the specific regulatory and organizational context of physiotherapy care in Germany. The concerns about lower treatment quality and inadequate care should be interpreted carefully. They may reflect legitimate clinical limitations of teletherapy for some indications, especially where hands-on assessment or manual techniques are central to treatment. However, they may also partly reflect limited exposure, uncertainty about appropriate indications, and insufficient training. Previous evidence suggests that real-time telerehabilitation can be effective and comparable to standard in-person care for several musculoskeletal conditions [20]. Therefore, the perceived inferiority of teletherapy in parts of the sample should not be interpreted as evidence of actual clinical inferiority. Rather, it points to the need for clearer indication criteria, clinical guidance, training, and examples of appropriate teletherapy use in physiotherapy.

### Interpretation of the Stratified Analyses

The exploratory descriptive stratified analyses add further insight into potential implementation patterns. Participants with prior teletherapy experience or use were more likely to consider teletherapy meaningful, more likely to feel sufficiently informed, and more likely to feel competent enough to offer teletherapy. They also reported key barriers such as integration into daily practice routines, insufficient legal or organizational information, and insufficient digital infrastructure less frequently than participants without prior experience. Although this subgroup was small and the findings should be interpreted cautiously, the pattern may suggest that practical exposure to teletherapy is associated with lower perceived uncertainty and higher self-assessed competence. Self-rated technical proficiency showed a similar, although less pronounced, pattern. Participants who rated themselves as very or somewhat technically proficient more often considered teletherapy meaningful and more often reported feeling competent enough to offer it. This supports the interpretation that digital confidence may be relevant for teletherapy adoption, but it also shows that technical proficiency alone is unlikely to be sufficient. Even among technically proficient participants, only a minority felt sufficiently informed. This reinforces the importance of legal, organizational, and clinical guidance in addition to technical skills. Professional experience showed a more complex pattern. Participants with 10 years or less of professional experience more often considered teletherapy meaningful for future care, but less often felt sufficiently informed or competent compared with those with more than 10 years of experience. They also reported several key barriers and facilitators more frequently. This may indicate that less experienced physiotherapists in this sample were open to teletherapy but perceived a greater need for structured support, training, and implementation guidance. Conversely, more experienced physiotherapists may feel more clinically confident but somewhat less convinced of teletherapy’s future role. These descriptive findings should not be overinterpreted. Because subgroup sizes were small for some strata, particularly the group with prior teletherapy experience, and because no inferential statistical testing was performed, the stratified analyses should be considered hypothesis-generating only.

### Implications for Practice, Policy, and Education

The findings have several implications for physiotherapy practice and health policy in Germany. First, professional associations and health insurance providers appear to have a central role in implementation. Participants most frequently expected these stakeholders to provide relevant information and support. This suggests that teletherapy implementation should not be left to individual practices alone. Physiotherapists may need clear, accessible, and practice-oriented information on legal requirements, reimbursement rules, documentation, data protection, suitable software, and clinical indications. This is consistent with the World Physiotherapy digital practice report, which emphasizes the need for coherent standards, education, and regulatory clarity to support safe and effective digital physiotherapy practice across countries [21]. Second, training should address both technical and clinical dimensions of teletherapy. Further training was the most frequently reported facilitator, and only 38.43% (176/458) of participants felt competent enough to work with teletherapy. Training should therefore not be limited to software use. It should also include patient selection, remote assessment, exercise instruction, risk management, communication skills in video-based care, documentation, and integration into existing workflows. In addition, teletherapy should not be treated as a homogeneous intervention. These considerations may be particularly relevant for musculoskeletal and chronic conditions, where education, exercise instruction, self-management support, and follow-up consultations are often central to physiotherapy care. In these areas, teletherapy may serve as a complementary mode of care when clinical safety, patient preferences, digital access, and the need for physical examination or hands-on treatment have been considered. Its suitability is likely to vary by clinical indication, treatment goal, patient preferences, safety considerations, and the extent to which assessment or treatment requires physical contact. Implementation guidance and training should therefore include indication-specific recommendations, for example, distinguishing between education, exercise instruction, self-management support, follow-up consultations, and interventions that require hands-on assessment or treatment. This is consistent with Rettinger and Kuhn’s [3] conclusion that infrastructure, education, training, guidelines, policies, and support systems are needed to improve telehealth services. Third, forthcoming policy changes may influence some of the reported implementation barriers. Governmental subsidies for hardware and software, the planned connection of physiotherapy practices to the telematics infrastructure, electronic prescriptions, and the electronic patient record may help reduce technical and organizational barriers and may increase familiarity with digital workflows in physiotherapy. However, these measures are unlikely to be sufficient on their own. The present findings suggest that infrastructure-related policies should be accompanied by clear reimbursement rules, legal and organizational guidance, clinical indication criteria, workflow support, and profession-specific training. Policy changes may therefore create more favorable conditions for teletherapy uptake, but future studies are needed to examine whether they translate into routine use. Because this study assessed perceptions, self-assessed competence, and reported barriers rather than actual implementation outcomes or longitudinal behavior, these policy implications should be interpreted as hypotheses for implementation support rather than as evidence that specific policy measures will increase teletherapy uptake. Fourth, physiotherapy education should prepare future professionals for hybrid models of care. The finding that less experienced participants were more likely to consider teletherapy meaningful but less likely to feel informed or competent points to a possible training gap. Teletherapy-related competencies could therefore be integrated into undergraduate and postgraduate physiotherapy education, including clinical reasoning for remote care, digital communication, safety screening, and ethical and legal aspects of digital practice.

### Contribution of This Study

This study contributes to the field by providing a current descriptive assessment of participating physiotherapists’ perceptions of teletherapy in Germany during a period of ongoing digital health policy development. While previous German studies have shown limited adoption of teletherapy or examined telerehabilitation in specific contexts, this study combines information on current and intended use, perceived barriers, facilitators, information needs, and self-assessed competence in a larger sample of physiotherapists. The study also adds exploratory stratified findings suggesting that prior teletherapy experience, technical proficiency, and professional experience may shape perceptions of teletherapy implementation. These findings can inform future implementation strategies, educational programs, and stakeholder guidance, while acknowledging the descriptive and nonrepresentative nature of the data.

### Limitations

Several limitations should be acknowledged. First, the open online survey design and the use of convenience sampling entail a risk of self-selection bias and prevent calculation of a conventional response rate. Because the survey link was disseminated through open professional and academic channels, the number of individuals who received or viewed the invitation could not be determined. Therefore, the sample cannot be assumed to be representative of all physiotherapists practicing in Germany, and the findings should not be generalized to the entire German physiotherapy workforce without caution. Second, the proportion of incomplete questionnaires was high. Of 761 individuals who started the survey, 458 completed it in full and were included in the analysis. Although available partial responses from incomplete questionnaires were reviewed descriptively, a comprehensive attrition analysis was not possible because demographic characteristics were collected at the end of the questionnaire and were largely unavailable for noncompleters. In addition, partial responses were available only for questionnaire sections reached before dropout. Therefore, selection bias cannot be ruled out, and the findings should be interpreted as reflecting the views of the completed respondent sample rather than as fully representative of all physiotherapists who initially accessed the survey. Third, sex and several practice-related characteristics, such as practice type, practice size, employment status, and care setting, were not assessed in the questionnaire. Consequently, the sample cannot be described with regard to sex distribution or these practice characteristics, and potential subgroup differences in perceptions, barriers, or implementation needs could not be examined. Fourth, the cross-sectional design limits causal interpretation. The findings describe reported perceptions, barriers, facilitators, and self-assessed competence at one point in time and cannot determine causal relationships or predict actual future implementation behavior. In addition, no inferential statistical testing was performed. Therefore, observed differences in the exploratory stratified analyses should be interpreted as descriptive patterns only and not as statistically confirmed group differences. Fifth, the study used a self-developed questionnaire that was pretested but not psychometrically validated. Therefore, measurement reliability could not be formally established, and measurement bias cannot be ruled out. In addition, constructs such as self-assessed competence, perceived meaningfulness, and perceived barriers were assessed as self-reported survey items rather than as theory-based or psychometrically validated scales. This limits the interpretation of these constructs and comparability with studies using validated instruments or different operationalizations of teletherapy-related barriers, facilitators, and competencies. The survey did not assess teletherapy suitability, barriers, or facilitators separately for specific clinical indications, patient groups, or treatment goals. Therefore, the findings cannot determine for which conditions or therapeutic purposes teletherapy may be appropriate. Finally, the study reflects only the perspective of physiotherapists. Patient perspectives, organizational decision-makers, health insurance providers, and software providers were not included, although these stakeholders are likely to influence the implementation of physiotherapeutic teletherapy in routine care. Therefore, conclusions about system-level implementation barriers should be interpreted as physiotherapists’ perceptions rather than as a comprehensive assessment of all relevant stakeholder perspectives.

### Conclusions

Physiotherapeutic teletherapy in Germany appears to be at an early stage of implementation in this sample. Although most participating physiotherapists considered teletherapy meaningful for future care, prior use was low and many respondents reported insufficient information, limited self-assessed competence, and practical barriers to implementation. The findings suggest that the central challenge may not simply be to convince physiotherapists of the relevance of teletherapy, but to create conditions that make teletherapy clinically appropriate, legally clear, technically feasible, and practically manageable in routine care. To support sustainable implementation, professional associations, health insurance providers, educational institutions, software providers, and policymakers should consider coordinated guidance, training opportunities, and implementation frameworks tailored to physiotherapy practice. Such measures should address not only digital infrastructure, but also clinical decision-making, workflow integration, reimbursement, data protection, and professional competencies. Future research should examine implementation strategies in real-world physiotherapy settings and include patient perspectives, organizational stakeholders, and longitudinal designs to assess whether positive attitudes can be translated into routine and sustainable teletherapy use.

## Supplementary material

10.2196/93733 Multimedia Appendix 1 Questionnaire for the online survey on physiotherapeutic teletherapy.

10.2196/93733 Checklist 1 CHERRIES checklist.

## Abbreviations

CHERRIES: Checklist for Reporting Results of Internet E-Surveys
DGPTW: German Society for Physiotherapy Science
DVMT: German Association for Musculoskeletal Therapy
GDPR: General Data Protection Regulation
