# Supplementary material for: Use and Perceptions of Teletherapy Among Physiotherapists in Germany: Descriptive Cross-Sectional Online Survey
Source: JMIR Form Res. 2026 Sep 21;10:e93733. doi: 10.2196/93733 (PMC13592741; doi:10.2196/93733)
Supplement: Supplementary file 1 [file formative-v10-e93733-s001.pdf]

Multimedia Appendix 2

Questionnaire for the Online Survey on Physiotherapeutic Teletherapy

Note. The questionnaire was administered in German using LimeSurvey. This appendix provides the original German item wording and an English translation. All survey items were mandatory. Where an "Other" option was provided, respondents could enter free text. Adaptive display logic was used where applicable, particularly for items related to current teletherapy use.

A. Previous experience and current use of physiotherapeutic teletherapy

Item 1

**Original German wording**	Nutzen Sie die Möglichkeit der physiotherapeutischen Teletherapie mit Ihren Patient*innen?
**English translation**	Do you use the option of physiotherapeutic teletherapy with your patients?
**Response format**	Single choice
**Response options**	• Ja / Yes<br>• Nein / No
**Note**	Used as an adaptive/filter question for subsequent items on current teletherapy use.

Item 2

**Original German wording**	An wie vielen Tagen im Monat nutzen Sie die physiotherapeutische Teletherapie?
**English translation**	On how many days per month do you use physiotherapeutic teletherapy?
**Response format**	Single choice with optional free-text "Other" response
**Response options**	• 1-5<br>• 6-10<br>• 11-15<br>• 16-20<br>• Über 20 / More than 20<br>• Sonstiges / Other
**Note**	Displayed to respondents who indicated use of physiotherapeutic teletherapy.

Item 3

**Original German wording**	Wie viele Patient*innen behandeln Sie pro Monat mithilfe der physiotherapeutischen Teletherapie?
**English translation**	How many patients do you treat per month using physiotherapeutic teletherapy?
**Response format**	Single choice with optional free-text "Other" response

**Response options**	• 1-5<br>• 6-10<br>• 11-20<br>• 21-30<br>• Über 30 / More than 30<br>• Sonstiges / Other
**Note**	Displayed to respondents who indicated use of physiotherapeutic teletherapy.

Item 4

**Original German wording**	Welche Software nutzen Sie für die Durchführung der physiotherapeutischen Teletherapie?
**English translation**	Which software do you use to conduct physiotherapeutic teletherapy?
**Response format**	Open text
**Response options**	Free-text or numeric response, as applicable.
**Note**	Displayed to respondents who indicated use of physiotherapeutic teletherapy.

Item 5

**Original German wording**	Beschreiben Sie positive wie negative Auffälligkeiten im Zusammenhang mit der Software.
**English translation**	Please describe positive and negative observations related to the software.
**Response format**	Open text
**Response options**	Free-text or numeric response, as applicable.
**Note**	Displayed to respondents who indicated use of physiotherapeutic teletherapy.

Item 6

**Original German wording**	Was war für Sie der Grund mit physiotherapeutische Teletherapie zu beginnen?
**English translation**	What was your reason for starting to use physiotherapeutic teletherapy?
**Response format**	Multiple choice with optional free-text “Other” response
**Response options**	• Eigenmotivation / Interesse an digitalen Möglichkeiten / Personal motivation or interest in digital options<br>• Monetärer Zugewinn / Financial gain<br>• Service für die Patient*innen / Service for patients<br>• Vorbereitung für zukünftige Versorgung / Preparation for future care<br>• Sie sehen keinen Grund dies zu tun / You see no reason to do this<br>• Sonstiges / Other

**Note**	Displayed to respondents who indicated use of physiotherapeutic teletherapy.

B. Barriers, facilitators, and support needs

Item 7

**Original German wording**	Was wäre für Sie ein Grund physiotherapeutische Teletherapie zukünftig zu nutzen?
**English translation**	What would be a reason for you to use physiotherapeutic teletherapy in the future?
**Response format**	Multiple choice with optional free-text "Other" response
**Response options**	• Eigenmotivation / Interesse an digitalen Möglichkeiten / Personal motivation or interest in digital options<br>• Monetärer Zugewinn / Financial gain<br>• Service für die Patient*innen / Service for patients<br>• Vorbereitung für zukünftige Versorgung / Preparation for future care<br>• Sie sehen keinen Grund dies zu tun / You see no reason to do this<br>• Sonstiges / Other
**Note**	Displayed depending on previous responses regarding current use.

Item 8

**Original German wording**	Was sind für Sie aktuelle Herausforderungen und Barrieren, die Ihre Nutzung oder geplante Nutzung der physiotherapeutischen Teletherapie erschweren oder verhindern?
**English translation**	What are current challenges and barriers that make it difficult or prevent you from using or planning to use physiotherapeutic teletherapy?
**Response format**	Multiple choice with optional free-text "Other" response
**Response options**	• Fehlendes Wissen im technischen Bereich / Lack of technical knowledge<br>• Fehlendes Wissen im fachlichen Bereich der Umsetzung von Teletherapie / Lack of professional knowledge regarding the implementation of teletherapy<br>• Informationen über die Rahmenbedingungen / Information on framework conditions<br>• Implementation in den Praxisalltag / Integration into daily practice<br>• Desinteresse der Patient*innen / Lack of patient interest<br>• Sie sehen keinen Mehrwert darin / You do not see any added value<br>• Fehlende Digitalisierung in der Praxis / Lack of digitalisation in the practice<br>• Fehlende oder nicht ausreichende Internetverbindung / No or insufficient internet connection<br>• Sonstiges / Other

Item 9

**Original German wording**	Fühlen Sie sich über die Möglichkeit der physiotherapeutischen Teletherapie ausreichend informiert, um sie zu nutzen?

**English translation**	Do you feel sufficiently informed about the possibility of physiotherapeutic teletherapy to use it?
**Response format**	Single choice
**Response options**	• Ja / Yes<br>• Nein / No

Item 10

**Original German wording**	Was hat Ihnen dabei geholfen die physiotherapeutische Teletherapie anzubieten bzw. was denken Sie würde Ihnen dabei helfen?
**English translation**	What has helped you to offer physiotherapeutic teletherapy, or what do you think would help you?
**Response format**	Multiple choice with optional free-text “Other” response
**Response options**	• Informationen durch Ihren Berufsverband / Information from your professional association<br>• Informationen durch die Krankenkasse / Information from health insurance providers<br>• Fortbildungen in diesem Bereich / Continuing education in this area<br>• Austausch/Hilfe durch Kolleg*innen / Exchange or support from colleagues<br>• Anregungen und Informationen durch Social Media (Facebook, Instagram, TikTok etc.) / Suggestions and information via social media (Facebook, Instagram, TikTok etc.)<br>• Sonstiges / Other

Item 11

**Original German wording**	Von wem würden Sie Hilfestellungen erwarten bzw. haben wollen?
**English translation**	From whom would you expect or want support?
**Response format**	Multiple choice with optional free-text “Other” response
**Response options**	• Berufsverband / Professional association<br>• Bundes-/Landesregierung / Federal or state government<br>• Krankenkassen / Health insurance providers<br>• Softwareanbieter / Software providers<br>• Sonstiges / Other

C. Perceived relevance, disadvantages, advantages, and competence

Item 12

**Original German wording**	Halten Sie physiotherapeutische Teletherapie für ein Konzept welches zukünftig in der Gesundheitsversorgung genutzt werden sollte?

**English translation**	Do you consider physiotherapeutic teletherapy to be a concept that should be used in future health care?
**Response format**	Single choice
**Response options**	• Ja / Yes<br>• Nein / No

Item 13

**Original German wording**	Was spricht für Sie gegen die Nutzung von physiotherapeutischer Teletherapie?
**English translation**	What, in your view, speaks against the use of physiotherapeutic teletherapy?
**Response format**	Multiple choice with optional free-text “Other” response
**Response options**	• Patient*innen benötigen immer direkten Kontakt zu Ihren Behandler*innen / Patients always require direct contact with their therapists<br>• Die Gefahr von Fehlversorgung ist zu hoch / The risk of inadequate care is too high<br>• Die Qualität der Behandlung ist schlechter als vor Ort / The quality of treatment is worse than in-person treatment<br>• Patient*innen wollen keine digitalen Behandlungen / Patients do not want digital treatments<br>• Die Vorgaben der Krankenkasse dazu sind noch nicht praktikabel / Health insurance requirements are not yet practicable<br>• Die verfügbare Software ist nicht gut genug / The available software is not good enough<br>• Sonstiges / Other

Item 14

**Original German wording**	Welche Vorteile sind für Sie relevant bei der Nutzung von physiotherapeutischer Teletherapie?
**English translation**	Which advantages are relevant to you when using physiotherapeutic teletherapy?
**Response format**	Multiple choice with optional free-text “Other” response
**Response options**	• Höhere Patientenanzahl in der gleichen Zeit zu behandeln / Treating a higher number of patients in the same amount of time<br>• Weniger Hausbesuche machen zu müssen / Having to conduct fewer home visits<br>• Keine Infektionsgefahr (für beide Seiten) / No risk of infection (for both sides)<br>• Verringerung des ökologischen Fußabdrucks (für beide Seiten) / Reduction of the ecological footprint (for both sides)<br>• Erweiterter Service für Patient*innen (positives Marketing) / Expanded service for patients (positive marketing)<br>• Sonstiges / Other

Item 15

**Original German wording**	Fühlen Sie sich kompetent genug physiotherapeutische Teletherapie anzubieten? Falls nein wählen Sie zutreffendes aus.

**English translation**	Do you feel competent enough to offer physiotherapeutic teletherapy? If not, please select all applicable options.
**Response format**	Multiple choice with optional free-text “Other” response
**Response options**	• Ja / Yes<br>• Nein, ich fühle mich nicht genügend ausgebildet im technischen Bereich / No, I do not feel sufficiently trained in the technical area<br>• Nein, ich fühle mich fachlich nicht in der Lage meine Therapie digital anzubieten / No, I do not feel professionally able to offer my therapy digitally<br>• Nein, ich bin über die Rahmenbedingungen nicht ausreichend informiert / No, I am not sufficiently informed about the framework conditions<br>• Sonstiges / Other

D. Competence requirements

Item 16

**Original German wording**	Welche Kompetenzen sind für Sie aus fachlicher Perspektive in der physiotherapeutischen Teletherapie anders gefordert als in der Physiotherapie vor Ort? Bitte begründen Sie Ihre Antwort kurz.
**English translation**	From a professional perspective, which competencies are required differently in physiotherapeutic teletherapy than in in-person physiotherapy? Please briefly justify your answer.
**Response format**	Open text
**Response options**	Free-text or numeric response, as applicable.
**Note**	These qualitative responses are not reported in the present manuscript and will be analysed and published separately.

E. Demographic and professional characteristics

Item 17

**Original German wording**	Wie alt sind Sie?
**English translation**	How old are you?
**Response format**	Numeric/open response
**Response options**	Free-text or numeric response, as applicable.

Item 18

**Original German wording**	Welcher ist Ihr höchster Abschluss im Bereich der Physiotherapie?

**English translation**	What is your highest qualification in the field of physiotherapy?
**Response format**	Single choice
**Response options**	• Staatsexamen / State examination<br>• Bachelorabschluss / Bachelor's degree<br>• Masterabschluss / Master's degree<br>• Promotion / Doctorate

Item 19

**Original German wording**	Wie viel Berufserfahrungen (in Jahren) haben Sie?
**English translation**	How much professional experience do you have (in years)?
**Response format**	Numeric/open response
**Response options**	Free-text or numeric response, as applicable.

Item 20

**Original German wording**	Wie viele Physiotherapeut*innen sind in der Praxis beschäftigt in der Sie tätig sind?
**English translation**	How many physiotherapists are employed in the practice where you work?
**Response format**	Numeric/open response
**Response options**	Free-text or numeric response, as applicable.

Item 21

**Original German wording**	Welcher Größenordnung ist die Stadt in der Ihre Praxis ist zuzuordnen, wählen Sie zutreffendes aus?
**English translation**	Which size category applies to the city or municipality where your practice is located? Please select the applicable option.
**Response format**	Single choice
**Response options**	• Städte und Gemeinden unter 5.000 Einwohner / Cities and municipalities with fewer than 5,000 inhabitants<br>• Ab 5.000 Einwohner (kleine Kleinstadt) / From 5,000 inhabitants (small town)<br>• Ab 10.000 Einwohner (große Kleinstadt) / From 10,000 inhabitants (large small town)<br>• Ab 20.000 Einwohner (kleine Mittelstadt) / From 20,000 inhabitants (small medium-sized city)<br>• Ab 50.000 Einwohner (große Mittelstadt) / From 50,000 inhabitants (large medium-sized city)<br>• Ab 100.000 Einwohner (kleinere Großstadt) / From 100,000 inhabitants (smaller large city)

	• Ab 500.000 Einwohner (große Großstadt) / From 500,000 inhabitants (large metropolitan city)

Item 22

**Original German wording**	Gibt es einen therapeutischen Schwerpunkt in der Praxis in der Sie tätig sind?
**English translation**	Is there a therapeutic focus in the practice where you work?
**Response format**	Single choice with optional free-text “Other” response
**Response options**	• Orthopädie / Orthopaedics<br>• Neurologie / Neurology<br>• Sportphysiotherapie / Sports physiotherapy<br>• Pädiatrie / Paediatrics<br>• Gemischtes Klientel / Mixed clientele<br>• Sonstiges / Other

Item 23

**Original German wording**	Würden Sie sich als technisch Affin einschätzen?
**English translation**	Would you describe yourself as technically inclined?
**Response format**	Single choice
**Response options**	• Ja, sehr / Yes, very much<br>• Ja, etwas / Yes, somewhat<br>• Neutral / Neutral<br>• Eher weniger / Rather less<br>• Gar nicht / Not at all
