# Supplementary material for: Use and Perceptions of Teletherapy Among Physiotherapists in Germany: Descriptive Cross-Sectional Online Survey
Source: JMIR Form Res. 2026 Sep 21;10:e93733. doi: 10.2196/93733 (PMC13592741; doi:10.2196/93733)
Supplement: Supplementary file 2 [file formative-v10-e93733-s002.docx]

**Checklist for Reporting Results of Internet E-Surveys (CHERRIES)**

| **Item Category** | **Checklist Item** | **Answer** |
| --- | --- | --- |
| Design | Describe survey design | The study was designed as a descriptive cross-sectional open online survey among practising physiotherapists in Germany who had completed professional training. The target population was estimated at approximately 200,000 physiotherapists in Germany. Based on this population size, a 95% confidence level, an assumed proportion parameter of p=.5, and a margin of error of +/-5%, the required sample size was calculated as approximately 384 participants. The study used a convenience sampling strategy. |
|  |  |  |
| IRB (Institutional Review  Board) approval and informed consent process | IRB approval | The study protocol was reviewed and approved by the Ethics Committee of Osnabrueck University of Applied Sciences before data collection (HSOS2024/1/15). |
|  | Informed consent | The landing page of the online survey provided information about the aim of the study, the voluntary nature of participation, the expected survey duration, data storage, and data processing. Participants provided informed consent before starting the questionnaire. The survey did not collect directly identifying personal information from participants. |
|  | Data protection | The survey was administered using LimeSurvey. Data were processed in accordance with the General Data Protection Regulation. The exported survey data were stored and analysed on a password-protected institutional laptop. Cookies/session identifiers were used to prevent or reduce multiple participation. |
|  |  |  |
| Development and pre-testing | Development and testing | The questionnaire was developed by the principal investigator based on the overarching research questions and the domains relevant to physiotherapeutic teletherapy. The questionnaire covered previous experience with teletherapy, barriers and challenges, facilitators and enabling factors, competencies required for teletherapy use, and demographic characteristics. A pretest was conducted with five physiotherapists with expertise in digital health care and questionnaire development. Feedback from the pretest was used to check the comprehensibility, relevance, and feasibility of the questionnaire. |
|  |  |  |
| Recruitment process and description  of the sample having access to the questionnaire | Open survey versus closed survey | The survey was conducted as an open survey. All individuals with access to the survey link were able to participate. |
|  | Contact mode | Potential participants were contacted through physiotherapy-related social media channels, alumni mailing lists of physiotherapy degree programmes, professional mailing lists, and an article on physio.de. |
|  | Advertising the survey | The survey was disseminated through the social media channels of the German Society for Physiotherapy Science (DGPTW), alumni mailing lists of the physiotherapy degree programmes at Osnabrueck University of Applied Sciences and Bremen University of Applied Sciences, the email distribution list of the German Association for Musculoskeletal Therapy (DVMT), and an article published on physio.de. The survey link was publicly accessible to individuals who received or accessed the recruitment information. |
|  |  |  |
| Survey administration | Web/E-mail | The survey was web-based and administered using LimeSurvey. Participation was possible via the survey link. The number of participants who started and completed the survey was recorded. |
|  | Context | The questionnaire was hosted on LimeSurvey, a platform used for online surveys. The context in which participants encountered the survey depended on the dissemination channel, including professional social media channels, alumni mailing lists, professional email lists, and physio.de. |
|  | Mandatory/voluntary | Participation in the survey was voluntary. |
|  | Incentives | No incentives or compensation were offered for participation. |
|  | Time/Date | Data collection took place from March 7, 2025, to May 21, 2025. |
|  | Randomization of items or questionnaires | Items were not randomized because the questionnaire was structured thematically and some questions built on previous responses. |
|  | Adaptive questioning | Adaptive questioning was used where applicable. Depending on previous responses, participants were shown relevant follow-up questions. |
|  | Number of Items | The questionnaire consisted of 23 items. |
|  | Number of screens (pages) | The questionnaire consisted of 12 screens/pages. |
|  | Completeness check | Survey progress was displayed to participants as a percentage. All questions had to be answered before participants could proceed. Where predefined response options were not suitable, an "Other" response option was available. Only fully completed questionnaires were included in the analysis. |
|  | Review step | Participants were able to return to previous pages and review or change their responses before final submission of the questionnaire. |
|  |  |  |
| Response rates | Unique site visitor | The number of unique site visitors was not reported. Cookies/session information was used to prevent or reduce repeated participation attempts. |
|  | View rate (Ratio of unique survey  visitors/unique site visitors) | The view rate was not reported because the survey was disseminated through open recruitment channels and the denominator of all individuals who viewed the invitation could not be determined. |
|  | Participation rate (Ratio of  unique visitors who agreed to  participate/unique first survey  page visitors) | The participation rate was not reported because the survey was disseminated through open recruitment channels and the number of individuals who viewed the first survey page or received the survey invitation could not be determined. |
|  | Completion rate (Ratio of users  who finished the survey/users  who agreed to participate) | A total of 761 individuals started the questionnaire, and 458 completed it in full and were included in the analysis. The completion rate was therefore 60.18%. |
|  |  |  |
| Preventing multiple entries from  the same individual | Cookies used | Cookies/session identifiers were used to prevent or reduce multiple participation. If the same browser attempted to access the survey again, LimeSurvey could identify the repeated access attempt using a session identifier and/or cookie. |
|  | IP check | IP addresses were not used for duplicate prevention. The manuscript reports the use of cookies/session identifiers to prevent or reduce multiple participation. |
|  | Log file analysis | No additional log file analysis was conducted to identify duplicate entries. |
|  | Registration | Registration was not required because the survey was conducted as an open survey. |
|  |  |  |
| Analysis | Handling of incomplete questionnaires | Only fully completed questionnaires were included in the analysis. Incomplete questionnaires were excluded. |
|  | Questionnaires submitted with  an atypical timestamp | Submission time did not affect inclusion in the analysis. Only completeness was used as an inclusion criterion for the final dataset. The questionnaire was designed to take approximately 10 minutes to complete. |
|  | Statistical correction | No weighting of items or other statistical correction procedures were applied. |
